# Comparison of *In Vitro* Hair Growth Promotion and Anti-Hair Loss Potential of Thai Rice By-Product from *Oryza sativa* L. cv. Buebang 3 CMU and Sanpatong

**DOI:** 10.3390/plants13213079

**Published:** 2024-11-01

**Authors:** Anurak Muangsanguan, Warintorn Ruksiriwanich, Chaiwat Arjin, Sansanee Jamjod, Chanakan Prom-u-Thai, Pensak Jantrawut, Pornchai Rachtanapun, Patipan Hnorkaew, Apinya Satsook, Mathukorn Sainakham, Juan Manuel Castagnini, Korawan Sringarm

**Affiliations:** 1 Department of Pharmaceutical Sciences, Faculty of Pharmacy, Chiang Mai University, Chiang Mai 50200, Thailand; anurak_m@cmu.ac.th (A.M.); pensak.j@cmu.ac.th (P.J.); mathukorn.s@cmu.ac.th (M.S.); 2 Center of Excellence in Agro Bio-Circular-Green Industry (Agro BCG), Agro-Industry, Chiang Mai University, Chiang Mai 50100, Thailand; pornchai.r@cmu.ac.th; 3 Department of Animal and Aquatic Sciences, Faculty of Agriculture, Chiang Mai University, Chiang Mai 50200, Thailand; chaiwat.arjin@cmu.ac.th; 4 Department of Plant and Soil Sciences, Faculty of Agriculture, Chiang Mai University, Chiang Mai 50200, Thailand; sansanee.j@cmu.ac.th (S.J.); chanakan.p@cmu.ac.th (C.P.-u.-T.); 5 Lanna Rice Research Center, Chiang Mai University, Chiang Mai 50200, Thailand; 6 School of Agro-Industry, Faculty of Agro-Industry, Chiang Mai University, Chiang Mai 50100, Thailand; 7 Office of Research Administration, Chiang Mai University, Chiang Mai 50200, Thailand; patipan.hnokaew@cmu.ac.th (P.H.); apinya.satsook@cmu.ac.th (A.S.); 8 Research Group in Innovative Technologies for Sustainable Food (ALISOST), Department of Preventive Medicine and Public Health, Food Science, Toxicology and Forensic Medicine, Faculty of Pharmacy, Universitat de València, Avenida Vicent Andrés Estellés s/n, 46100 Burjassot, Spain; juan.castagnini@uv.es

**Keywords:** rice bran (*Oryza sativa* L.), Buebang 3 CMU, Sanpatong, androgenetic alopecia, 5α-reductase inhibition, hair growth promotion, Wnt/β-catenin, Sonic Hedgehog, angiogenesis

## Abstract

The bioactive compounds in herbal extracts may provide effective hair loss treatments with fewer side effects compared to synthetic medicines. This study evaluated the effects of Buebang 3 CMU and Sanpatong rice bran extracts, macerated with dichloromethane or 95% ethanol, on hair growth promotion and hair loss prevention. Overall, Buebang 3 CMU extracts contained significantly higher levels of bioactive compounds, including *γ*-oryzanol, tocopherols, and various polyphenols such as phytic acid, ferulic acid, and chlorogenic acid, compared to Sanpatong extracts. Additionally, ethanolic extracts demonstrated greater bioactive content and antioxidant activities than those extracted with dichloromethane. These compounds enhanced the proliferation of human hair follicle dermal papilla cells (HFDPCs) by 124.28 ± 1.08% (*p* < 0.05) and modulated anti-inflammatory pathways by reducing nitrite production to 3.20 ± 0.36 µM (*p* < 0.05). Key hair growth signaling pathways, including Wnt/β-catenin (CTNNB1), Sonic Hedgehog (SHH, SMO, GLI1), and vascular endothelial growth factor (VEGF), were activated by approximately 1.5-fold to 2.5-fold compared to minoxidil. Also, in both human prostate cancer (DU-145) and HFDPC cells, the ethanolic Buebang 3 CMU extract (Et-BB3-CMU) suppressed *SRD5A1*, *SRD5A2*, and *SRD5A3* expression—key pathways in hair loss—by 2-fold and 1.5-fold more than minoxidil and finasteride, respectively. These findings suggest that Et-BB3-CMU holds promise for promoting hair growth and preventing hair loss.

## 1. Introduction

Androgenetic alopecia (AGA), commonly known as pattern hair loss, is the most common type characterized by progressive terminal hair loss after midlife. The hair cycle consists of four main stages: anagen (growth), catagen (regression), telogen (resting), and exogen (shedding), each influenced by various factors. The primary risk factor for AGA is the androgen hormone [1]. Dihydrotestosterone (DHT), an androgenic steroid hormone, is produced by the conversion of testosterone (TT) to DHT through the action of 5α-reductase (SRD5A) enzymes. Elevated levels of DHT in hair follicles can shorten the anagen stage, leading to follicle shrinkage and eventual hair loss [2]. Additionally, inflammation of hair follicles has been identified as a potential pathogenic factor in AGA. Nitric oxide (NO) levels in hair follicles are increased in response to oxidative stress and DHT [3]. Conversely, the hair growth development requires the interaction between mesenchymal, epithelial, and fibroblast cells in the hair follicle, which receive signaling from human hair follicle dermal papilla cells (HFDPC), such as Wnt/β-catenin (CTNNB1), Sonic Hedgehog (SHH, SMO, and GLI1), and vascular endothelial growth factor (VEGF) signaling pathways. The Wnt/β-catenin is the major pathway of hair growth development. The activation of β-catenin in HFDPC cells at the hair follicles stimulates the lymphoid enhancer factor (LEF) or T-cell factor (TCF) complex and activates the transcription of downstream target genes such as c-Myc and cyclin D1. This mechanism promotes the proliferation and migration of HFDPC cells in the initial stages of the growth stage of the hair cycle [4]. Furthermore, Wnt/β-catenin acts as an upstream regulator of the Sonic Hedgehog signaling pathway, which promotes the development of hair follicles in the middle stage of the growth phase of the hair cycle by stimulating the proliferation and migration of epithelial, mesenchymal, and fibroblast cells [4,5]. Moreover, VEGF induces angiogenesis in the growth phase and increases the supply of nutrients and oxygen-rich blood to the hair follicle [6].

Currently, several treatments for AGA, including topical minoxidil, oral finasteride, platelet-rich plasma (PRP) therapy, and low-level laser therapy have been applied [7]. Minoxidil is widely used for treating AGA in both males and females, primarily through mechanisms such as promoting angiogenesis by stimulating the expression of *VEGF* [7]. Finasteride, an inhibitor of the SRD5A type 2 isoenzyme, is originally used for treating benign prostatic hyperplasia and is also effective in treating AGA [8]. However, these drugs are associated with side effects such as skin irritation, burning sensations, impotence, decreased libido, sexual dysfunction, and gynecomastia [9]. These limitations have led to growing interest in plant-based anti-hair loss agents and hair growth-promoting substances as alternative, non-invasive treatments for hair growth. Various plants are rich in bioactive compounds, such as polyphenols, terpenoids, carotenoids, and fatty acids, which are known to promote hair growth. Traditionally, plants like turmeric (*Curcuma longa*), rice (*Oryza sativa*), shallot (*Allium ascalonicum*), and butterfly pea (*Clitoria ternatea*) have been used to treat hair loss [10,11,12,13].

The staple food rice (*Oryza sativa* L.) is extensively consumed on a global scale. Buebang 3 CMU and Sanpatong are two different varieties of rice found in Thailand. The majority of them are cultivated in the northern part of Thailand. The non–glutinous local rice Buebang 3 CMU was developed by breeding at the Lanna Rice Research Institute, Chiang Mai University, Thailand. Whereas Sanpatong rice was a popular glutinous rice in Chiang Mai. A typical rice composition consists of rice husk, rice barn, brown rice, polished rice, or milled rice [14]. Rice bran is a major byproduct of rice milling, accounting for around 8–10% of the rice grain [15]. Typically, rice barns were commonly utilized for animal feed, fertilizer, or fuel generation. Rice bran refers to the extent to which the germ and bran layers of the brown rice kernel have been removed during the polishing process in order to produce white rice [15,16,17]. Numerous studies have documented that rice bran contains various kinds of bioactive components, such as polyphenols, phenolic compounds (flavonoids and *γ*-oryzanol), polysaccharides, and tocopherols [5,6,7,8]. According to Wisetkomolmat et al. [15], Buebang 3 CMU rice bran has a significant amount of *γ*-oryzanol (219 mg/100 g crude fat) and several phenolic compounds, including phytic acid (3.29 mg/100 g sample), *p*-coumaric acid (1.15 mg/100 g sample), and ferulic acid (0.18 mg/100 g sample). While Sanpatong rice barn showed a significant level of efficiency in terms of antioxidant activity, measuring 13.8 μmol Fe^2+^/g [18]. The functional characteristics of rice bran make it well-suited for use in commercial applications within the nutraceutical and pharmaceutical industries.

The effective utilization of rice by-products could provide benefits for farmers and decrease the environmental pollution caused by their disposal. While some studies have demonstrated differences in the antioxidant potential of various rice fractions, no previous research has thoroughly identified and compared rice bran extracts from different varieties and solvent extractions regarding their anti-inflammatory and antioxidant activities, as well as their effects on the expression of genes related to hair growth and hair loss [14,19]. The transformation of agricultural residues into valuable anti-hair loss products with the support of scientific evidence could introduce the alternative application for anti-hair loss market. Accordingly, the objective of this study is to compare the bioactive compounds and biological activities related to hair loss prevention and hair growth promotion between two rice bran varieties: Buebang 3 CMU (a non-glutinous rice) and Sanpatong (a glutinous rice). Previous research has shown that Buebang 3 CMU rice bran contains high levels of bioactive compounds and exhibits strong biological activities for preventing hair loss and promoting hair growth [11,14,15,20]. Sanpatong rice bran, whose rice grain is widely consumed in northern Thailand, was selected to compare its potential hair loss prevention and hair growth promotion to that of Buebang 3 CMU rice bran. Additionally, this study aims to evaluate the bioactive compounds in rice bran extracts from Buebang 3 CMU and Sanpatong varieties obtained from different solvent extractions (ethanol and dichloromethane) to determine their effects on antioxidant and anti-inflammatory activities, as well as the regulation of gene expression for *SRD5A1-3*, *CTNNB1*, *SHH*, *SMO*, *GLI1*, and *VEGF*, there by confirming the potential application of rice bran extract in the therapy of AGA.

## 2. Results and Discussion

### 2.1. Bioactive Compounds and Antioxidant Potentials

Table 1 illustrates the content of the percentage extraction yield, total phenolic, and flavonoid contents in rice bran extracts. In terms of percentage extraction yield, the Di-BB3-CMU extract exhibited the highest yield (14.85), followed by the Di-SPT (13.47), Et-SPT (7.32), and Et-BB3-CMU extracts (4.38), respectively. The Et-BB3-CMU extract showed the highest mg GAE/g extract of total phenolic content (6.60 ± 0.22), followed by the Et-SPT (6.40 ± 0.31), Di-SPT (4.10 ± 0.09), and Di-BB3-CMU extracts (3.87 ± 0.07), respectively. Whereas the total flavonoid content was highest in the Et-BB3-CMU extract (10.42 ± 0.11), followed by the Et-SPT (7.45 ± 0.15), Di-BB3-CMU (5.73 ± 0.15), and Di-SPT extracts (2.36 ± 0.04), respectively.

The *γ*-oryzanol and tocopherol contents of rice bran extracts are shown in Table 2. The rice bran extracts obtained using dichloromethane (Di-SPT and Di-BB3-CMU) had greater *γ*-oryzanol and tocopherol contents than the extracts obtained using ethanol as a solvent (Et-SPT and Et-BB3-CMU). Interestingly, the BB3-CMU rice bran extracts displayed higher levels of *γ*-oryzanol, and tocopherol contents compared to the SPT rice bran extracts in both rice bran extracts obtained using ethanol and dichloromethane as a solvent. These results agreed with Khantham et al. [19] and Wisetkomolmat et al. [15], who reported that rice bran extracts (KDML105 and BB3-CMU) contain bioactive components including tocopherols and *γ*-oryzanol. Furthermore, previous research indicates that *γ*-oryzanol and tocopherol may inhibit the expression of the *SRD5A* gene, which plays a key role in hair loss [14,19]. Additionally, *γ*-oryzanol and tocopherol can stimulate hair growth pathways, including Wnt/β-catenin, Sonic Hedgehog, and the angiogenesis pathway. These mechanisms contribute to prolonging the anagen phase of the hair cycle and promoting new hair regeneration [19].

The antioxidant potentials using the DPPH and ABTS methods of BB3-CMU and SPT rice bran extracts were slightly different from those reported previously [11,18], this may be because of the extraction methods or different durations, which may have altered bioactive compound content and antioxidant activity. For antioxidant potentials using the FRAP method, the rice bran extracts obtained using ethanol as a solvent (Et-SPT and Et-BB3-CMU) had greater antioxidant potentials than the extracts obtained using dichloromethane (Di-SPT and Di-BB3-CMU), as shown in Table 3. The antioxidant potentials of both Et-SPT and Et-BB3-CMU agreed with those of the high total phenolic and total flavonoid contents in the rice bran extracts. The total phenolic and total flavonoid contents are considered indicators of the antioxidant capacity since the redox properties of the phenolic compounds allow them to exhibit as reducing agents via hydrogen donors and radical scavengers [21]. High levels of antioxidants may reduce the oxidative stress associated with androgenetic alopecia (AGA) and delay the premature aging of hair follicles. In HFDPCs, excessive accumulation of reactive oxygen species (ROS) can exceed their antioxidative capacity, leading to premature senescence [22]. Previous research has shown that HFDPCs from AGA patients exhibit increased sensitivity to oxidative stress and external factors, such as pollution and UV radiation, compared to non-balding cells [23]. This heightened sensitivity results in reduced cell proliferation and migration of HFDPCs, ultimately affecting hair follicle development and hair growth.

### 2.2. Phenolic Compounds

The content of polyphenolic compounds in rice bran extracts is summarized in Table 4. Thirteen compounds were identified by comparison of their mass spectra and retention times of standards available in the laboratory. In the rice bran extracts, the phenolic compound with the highest content was phytic acid, with Et-BB3-CMU (13.43 ± 0.14 mg/g extract) having a higher content than Et-SPT (10.91 ± 0.03 mg/g extract). Our results were similar to those previously reported by Wisetkomolmat et al. (2022) [15], who found that phytic acid is the most abundant phenolic compound in different varieties of rice bran. Especially rice bran variety BB3-CMU. Gallocatechin gallate, caffeic acid, ferulic acid, and chlorogenic acid were also higher in the Et-BB3-CMU extract than in the Et-SPT extracts. Our results confirmed that rice bran contains an abundance of polyphenols. These bioactive compounds may impact molecular mechanisms associated with hair growth promotion, including antioxidant activities, anti-inflammatory effects, and modulation of androgenic pathways [14,19].

**Table 1:** Total phenolic and flavonoid contents of rice bran extracts.

Extracts	Yield (%)	Total Phenolic Content (mg GAE/g Extract)	Total Flavonoids Content (mg CE/g Extract)
Di-SPT	13.47	4.10 ± 0.09	2.36 ± 0.04
Di-BB3-CMU	14.85	3.87 ± 0.07	5.73 ± 0.15
Et-SPT	7.32	6.40 ± 0.31	7.45 ± 0.15
Et-BB3-CMU	4.38	6.60 ± 0.22	10.42 ± 0.11

Note: All values were expressed as the mean ± SD; mg GAE/g extract: mg of gallic acid equivalents per g of extract; mg CE/g extract: mg of catechin equivalents per g of extract; Di-SPT: Sanpatong rice bran extracts using dichloromethane as the solvent; Et-SPT: Sanpatong rice bran extracts using ethanol as the solvent; Di-BB3-CMU: Buebang 3-CMU rice bran extracts using dichloromethane as the solvent; Et-BB3-CMU: Buebang 3-CMU rice bran extracts using ethanol as the solvent.

**Table 2:** The *γ*-oryzanol and tocopherol contents of rice bran extracts.

Extracts	Compound Contents (mg/100 g Extract)			
	*γ*-Oryzanol	α-Tocopherol	*β* + *γ*-Tocopherol	δ-Tocopherol
Di-SPT	1322.10 ± 15.32	12.99 ± 0.02	8.93 ± 0.03	1.15 ± 0.01
Di-BB3-CMU	1752.75 ± 53.05	31.55 ± 0.74	9.06 ± 0.49	1.30 ± 0.01
Et-SPT	144.30 ± 2.88	5.08 ± 0.04	2.12 ± 0.02	0.63 ± 0.02
Et-BB3-CMU	152.49 ± 0.29	6.28 ± 0.04	3.35 ± 0.03	0.83 ± 0.01

Note: All values were expressed as the mean ± SD; Di-SPT: Sanpatong rice bran extracts using dichloromethane as the solvent; Et-SPT: Sanpatong rice bran extracts using ethanol as the solvent; Di-BB3-CMU: Buebang 3-CMU rice bran extracts using dichloromethane as the solvent; Et-BB3-CMU: Buebang 3-CMU rice bran extracts using ethanol as the solvent.

**Table 3:** Antioxidant potentials of rice bran extracts.

Extracts	DPPH Scavenging Activity (IC_50_, mg/mL)	ABTS Scavenging Activity (IC_50_, mg/mL)	FRAP Reducing Power (mM Fe^2+^/g Extract)
Di-SPT	36.08 ± 0.32	79.39 ± 0.67	63.91 ± 4.74
Di-BB3-CMU	40.52 ± 0.39	66.08 ± 2.79	55.45 ± 2.83
Et-SPT	9.65 ± 0.45	11.53 ± 0.35	119.71 ± 3.63
Et-BB3-CMU	10.62 ± 0.85	6.58 ± 0.57	88.46 ± 0.63

Note: All values were expressed as the mean ± SD; IC_50_: the 50% half maximal inhibitory concentration (mg/mL); mM Fe^2+^/g extract: µM ferrous ion per g of extract; Di-SPT: Sanpatong rice bran extracts using dichloromethane as the solvent; Et-SPT: Sanpatong rice bran extracts using ethanol as the solvent; Di-BB3-CMU: Buebang 3-CMU rice bran extracts using dichloromethane as the solvent; Et-BB3-CMU: Buebang 3-CMU rice bran extracts using ethanol as the solvent.

**Table 4:** Phenolic contents of rice bran extracts.

Items	Extracts (mg/g Extract)	
	Et-SPT	Et-BB3-CMU
Caffeic acid	5.55 ± 0.01	6.90 ± 0.01
Epicatechin	2.31 ± 0.02	2.18 ± 0.00
Gallocatechin gallate	6.93 ± 0.14	7.47 ± 0.13
*p*-oumaric acid	3.70 ± 0.01	3.61 ± 0.08
*o*-coumaric acid	2.99 ± 0.01	2.76 ± 0.01
Naringin	0.45 ± 0.00	0.55 ± 0.01
Rosmarinic acid	1.92 ± 0.01	ND
Quercetin	4.87 ± 0.03	3.99 ± 0.05
Rutin	2.36 ± 0.05	2.19 ± 0.00
Phytic acid	10.91 ± 0.03	13.43 ± 0.14
Ferulic acid	3.02 ± 0.01	3.35 ± 0.02
Chlorogenic acid	0.95 ± 0.02	1.04 ± 0.05
Hydroxybenzoic acid	1.84 ± 0.01	2.39 ± 0.06

Note: All values were expressed as the mean ± SD; ND: non detectable; Et-SPT: Sanpatong rice bran extracts using ethanol as the solvent; Et-BB3-CMU: Buebang 3-CMU rice bran extracts using ethanol as the solvent.

### 2.3. Effect of Rice Bran Extracts on Cell Viability

The anti-inflammatory, antioxidant activities, and effects on gene expression profiling of four rice bran extracts at concentrations ranging from 0.0625 to 2 mg/mL were evaluated in RAW 264.7, DU-145, and HFDPC cells. According to ISO 10993-5, a percentage of cell survival higher than 80% is regarded as non-toxic [24]. After 24 h of incubations, all rice bran extracts at a concentration of 0.0625–0.125 mg/mL for DU-145 and HFDPC cells demonstrated no cytotoxicity compared to untreated cells (*p* > 0.01). In RAW 264.7 cells, all rice bran extracts at a concentration above 0.250 mg/mL showed cytotoxicity and significantly improved cell survival compared to untreated cells. Thereby, the highest concentration of rice bran extracts of all cells (0.125 mg/mL) that gave the cell survival more than 80% was selected for further experiment.

In addition, the Et-SPT (121.52 ± 1.26%) and Et-BB3-CMU (124.28 ± 1.08%) rice bran extracts at a concentration of 0.0625 mg/mL significantly increased HFDPC cell viability compared to minoxidil (108.14 ± 0.86), which was used as a standard control in this study (*p* < 0.05). Previous studies have reported that minoxidil promotes the proliferation of HFDPCs by activating the extracellular signal-regulated kinase (ERK) and protein kinase B (AKT) signaling pathways. Furthermore, minoxidil prevents apoptosis in HFDPCs by increasing the ratio of B-cell lymphoma 2 (BCL-2) to BCL-2-associated X protein (Bax), both of which are key regulators of pro- and anti-apoptotic activities [25]. HFDPC cells, located at the base of the hair follicle, are crucial for hair follicle formation and the initiation of hair growth. The interaction between HFDPCs and other cell types, such as epidermal and fibroblast cells within the hair follicle, plays a significant role in the hair cycle, particularly during the anagen phase. Therefore, the observed proliferation of HFDPC cells in response to Et-SPT and Et-BB3-CMU rice bran extracts in this study suggests a potential role in supporting hair follicle formation and the initiation of hair growth [26].

### 2.4. Effect of Rice Bran Extracts on Anti-Inflammatory Activities

**Figure 1:**
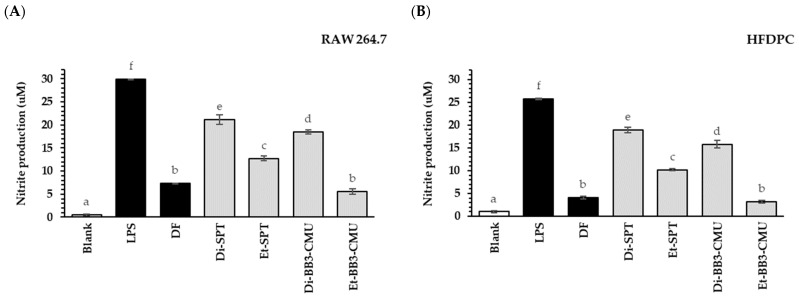
The effects of rice bran extracts and the standard control (diclofenac sodium) at a concentration of 0.125 mg/mL on nitrite production in lipopolysaccharide (LPS)-induced RAW 264.7 (**A**) and HFDPC (**B**) cells after 24 h, compared to a solvent-treated control without LPS (blank) and an LPS-induced control (+LPS). DF: diclofenac sodium; Di-SPT: Sanpatong rice bran extracts using dichloromethane as the solvent; Et-SPT: Sanpatong rice bran extracts using ethanol as the solvent; Di-BB3-CMU: Buebang 3-CMU rice bran extracts using dichloromethane as the solvent; Et-BB3-CMU: Buebang 3-CMU rice bran extracts using ethanol as the solvent. Values are expressed as the mean ± SD for triplicate samples. Statistical analysis was performed using one-way ANOVA followed by Tukey’s HSD test. Different letters (a–f) indicate statistically significant differences (*p* < 0.05) between samples.

Nitric oxide synthase or iNOS is induced by noxious circumstances, including injury, infection, oxidative stress, and androgen [27]. As a result, perifollicular macrophages produce and release large amounts of nitric oxide (NO), leading to increased inflammation and tissue damage. Notably, stimulation of HFDPCs with DHT resulted in a three-fold increase in NO levels, mediated by inducible iNOS [27,28]. Perifollicular inflammation and inflammatory infiltration are recognized as histological characteristics of pattern hair loss [29]. In this study, the rice bran extracts and diclofenac sodium (DF) at the same non-toxicity concentration (0.125 mg/mL) were determined on RAW 264.7 and HFDPC cells to compare the inhibitory effect on nitric oxide (NO) production. As shown in Figure 1, the NO levels in the LPS-treated group, without any pretreatment, were higher than those in the solvent-treated control group (blank) in both RAW 264.7 and HFDPC cells. Furthermore, all tested samples significantly reduced nitrite production compared to the nitrite levels in the LPS-treated group (*p* < 0.05). For the overall results, Et-BB3-CMU showed significantly greater nitrite suppression compared to other samples. Notably, there was no significant difference in nitrite levels between Et-BB3-CMU and diclofenac (a standard anti-inflammatory drug) in both RAW 264.7 and HFDPC cells (*p* < 0.05). In comparing the solvent extraction methods, the result indicated that the rice bran extract obtained using ethanol as a solvent had a greater reduced nitrite production than the extract obtained using dichloromethane in both RAW 264.7 and HFDPC cells. Previous studies reported that NO can regulate various aspects of hair biology [30]. A combination of several plant extracts in the hair tonic formulation has been demonstrated to induce hair growth promotion and decrease inflammation by suppressing iNOS and transforming growth factor beta (TGF-β) in hair follicles [31]. Polyphenols in rice bran are known for their anti-inflammatory properties, primarily due to their ability to scavenge free radicals and suppress pro-inflammatory enzymes such as lipoxygenase (LOX), cyclooxygenase-2 (COX-2), and inducible nitric oxide synthase (iNOS) [32]. In our study, the rice bran extracts obtained using ethanol as the solvent (Et-SPT and Et-BB3CMU) showed significantly greater nitrite suppression compared to those using dichloromethane as the solvent (Di-SPT and Di-BB3CMU) (*p* > 0.05). This finding aligns with previous research demonstrating that the ethanolic extracts contained higher polyphenol content than the dichloromethane extracts [33]. Additionally, our results suggest that the synergistic effects of polyphenols present in rice bran extracts—such as caffeic acid, epicatechin, *p*-coumaric acid, *o*-coumaric acid, naringin, rosmarinic acid, quercetin, rutin, phytic acid, and ferulic acid—contributes to their anti-inflammatory and antioxidant activities [14,34]. As shown, the BB3-CMU rice bran extracts which exhibited significant higher levels of polyphenols, (*γ*-oryzanol, and tocopherol) compared to the SPT rice bran extract, also exhibited the higher nitric oxide suppression. This finding aligns with previous research showing that BB3-CMU rice bran significantly reduces inflammation by suppressing NO production [14].

### 2.5. Effect of Rice Bran Extracts on Antioxidant Activities in HFDPC Cells

**Figure 2:**
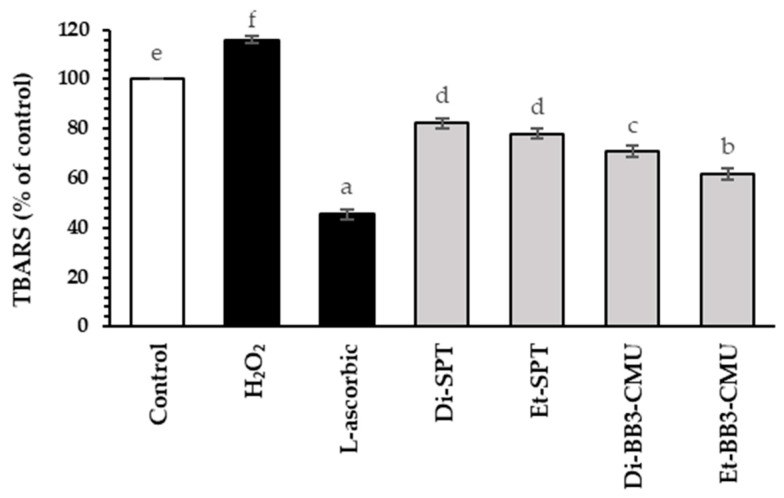
Effects of rice bran extracts and standard controls (L-ascorbic acid) at the concentration of 0.125 mg/mL on the malondialdehyde production in hydrogen peroxide (H_2_O_2_)-induced HFDPCs using the thiobarbituric acid reactive substances (TBARS) assay. Di-SPT: Sanpatong rice bran extracts using dichloromethane as the solvent; Et-SPT: Sanpatong rice bran extracts using ethanol as the solvent; Di-BB3-CMU: Buebang 3-CMU rice bran extracts using dichloromethane as the solvent; Et-BB3-CMU: Buebang 3-CMU rice bran extracts using ethanol as the solvent. Values were expressed as the mean ± SD for triplicates in each sample. Statistical analysis was performed using a one-way ANOVA, followed by Tukey’s HSD test. Different letters (a–f) indicate statistical differences (*p* < 0.05) in comparison to each sample.

Malondialdehyde is a by-product of the lipid peroxidation process. The reaction between malondialdehyde and thiobarbituric acid produces a dark pink compound that can be quantified using a UV-Vis spectrophotometer [35]. The thiobarbituric acid reactive substances (TBARS) method was used to evaluate the malondialdehyde production in hydrogen peroxide (H_2_O_2_)-induced HFDPC cells, as demonstrated in Figure 2. The antioxidant activities of the rice bran extracts were compared to L-ascorbic acid (45.51 ± 2.08% of control), which served as a positive control in this assay. The TBARS quantification for H₂O₂-stimulated HFDPC cells (115.94 ± 1.03% of control) was significantly different from that of the control group (*p* < 0.05). Additionally, all rice bran extracts (Di-SPT, Et-SPT, Di-BB3-CMU, and Et-BB3-CMU) exhibited antioxidant activities, with suppressed TBARS quantifications of 82.28 ± 2.08%, 78.08 ± 2.00%, 70.75 ± 2.31%, and 61.84 ± 2.31% of control, respectively. Furthermore, the ethanoic rice bran extracts (Et-SPT and Et-BB3-CMU) had a greater suppression of TBARS quantification than the dichloromethane extract (Di-SPT and Di-BB3-CMU). This may be due to the potent antioxidant properties of polyphenol content in ethanolic extracts [36]. Zhang et al. found that mulberry, which is rich in polyphenols and anthocyanins, exhibited strong antioxidant activities [37]. Polyphenols can inhibit the oxidation reaction by donating hydrogen atoms to radical species and convert into phenoxyl radicals. This finding is consistent with previous research showing that polyphenols can slow down lipid oxidation by interrupting radical chain reactions [38].

### 2.6. Effects of Rice Bran Extracts on Genes Expression

**Figure 3:**
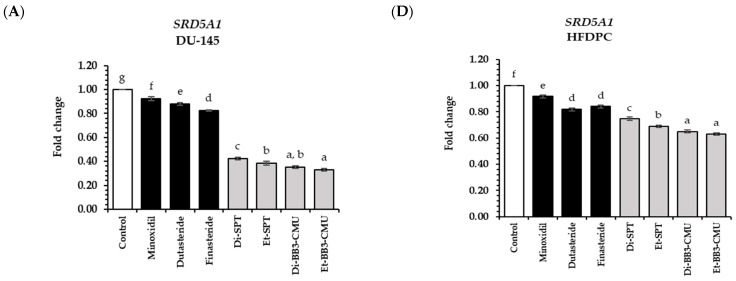

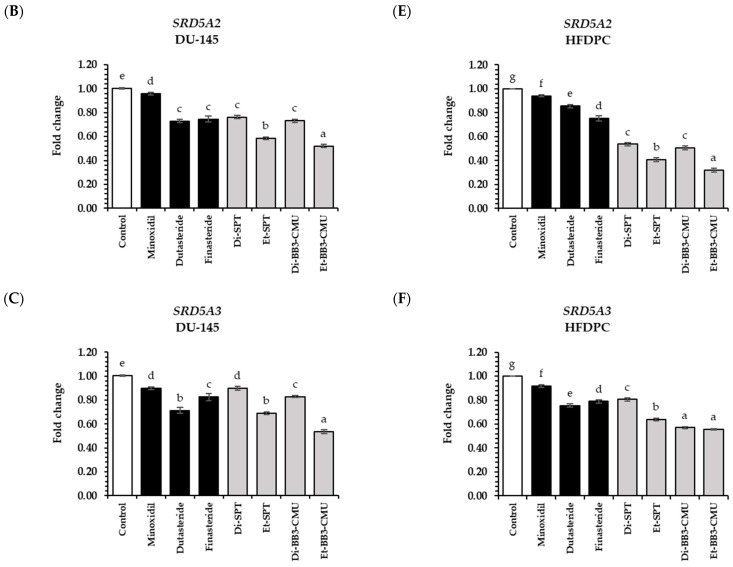
The effects of rice bran extracts on the gene expression in the androgen pathway; (**A**) *SRD5A1*, (**B**) *SRD5A2*, and (**C**) *SRD5A3* in DU-145 cells; and (**D**) *SRD5A1*, (**E**) *SRD5A2*, and (**F**) *SRD5A3* in HFDPCs were compared to the standard controls (minoxidil, dutasteride, and finasteride) at a concentration of 0.125 mg/mL. Di-SPT: Sanpatong rice bran extracts using dichloromethane as the solvent; Et-SPT: Sanpatong rice bran extracts using ethanol as the solvent; Di-BB3-CMU: Buebang 3-CMU rice bran extracts using dichloromethane as the solvent; Et-BB3-CMU: Buebang 3-CMU rice bran extracts using ethanol as the solvent. The results were shown as a fold change in gene expression relative to the control (untreated). Statistical analysis was performed using one-way ANOVA, followed by Tukey’s HSD test. Different letters (a–g) within each sample indicate significant differences (*p*-value < 0.05).

Hair growth is a complex process comprising several stages of the hair cycle, including anagen (growth), catagen (regression), telogen (resting), and exogen (shedding), all of which are influenced by various factors [39]. This study identified the major regulatory pathways involved in hair growth development, including the androgen, Wnt/β-catenin, Sonic Hedgehog, and angiogenesis signaling pathways [40]. The androgen pathway, in particular, involves dihydrotestosterone (DHT), an androgenic steroid hormone that is produced by the action of SRD5A enzymes, which convert testosterone to DHT. Elevated levels of DHT in hair follicles can shorten the anagen phase of the hair cycle, leading to premature hair follicle shrinkage and subsequent hair loss [2]. Rice bran contains various compounds, including polyphenols, tocopherol, and oryzanol [15]. Previous studies showed that polyphenols, tocopherol, and oryzanol in rice bran played a crucial role in *SRD5A* gene suppression [19]. In order to evaluate the regulatory effect of rice bran extracts (0.125 mg/mL) on the expressions of genes encoding *SRD5A1*, *SRD5A2*, and *SRD5A3* associated with the androgen pathway in DU-145 and HFDPC cells. The rice bran extracts and standard controls, such as minoxidil, dutasteride, and finasteride, were determined at the same concentration of 0.125 mg/mL. The suppression effects of rice bran extracts on the genes associated with the androgen pathway are shown in Figure 3.

Both in DU-145 and HFDPC cells, all of the rice bran extracts significantly decreased the expression of *SRD5A1*, *SRD5A2*, and *SRD5A3* genes compared to untreated cells (control group) (*p* < 0.05). As a result, the Et-BB3-CMU extract indicated the highest *SRD5A1-3* gene suppression, with a fold change of 0.33 ± 0.01, 0.52 ± 0.01, and 0.54 ± 0.01 in DU-145 cells and 0.63 ± 0.01, 0.32 ± 0.02, and 0.56 ± 0.01 in HFDPC cells, respectively. Moreover, the Et-BB3-CMU extract exhibited significantly 2 and 1.5 times higher activity than the standard minoxidil and finasteride, respectively, in both DU-145 and HFDPC cells (*p* < 0.05). This effect is likely due to the high polyphenol content in Et-BB3-CMU, particularly phytic acid (13.43 ± 0.14 mg/g extractgallocatechin gallate (7.47 ± 0.13), gallocatechin gallate (7.47 ± 0.13 mg/g extract), and caffeic acid (6.90 ± 0.01 mg/g extract), which exhibited their strong free radical scavenging activity [14,41]. These findings are consistent with previous research suggesting that these polyphenols can prevent hair loss by suppressing the 5α-reductase enzyme [41,42].

At the same solvent extraction method, the BB3-CMU rice bran extract exhibited stronger suppression of *SRD5A1-3* gene expression and significantly higher activity compared to the SPT rice bran extract (*p* > 0.05). This aligns with the significant differences observed in the bioactive compound levels, with BB3-CMU rice bran containing higher amounts of polyphenols, *γ*-oryzanol, and tocopherol than SPT rice bran (*p* > 0.05) [15].

The rice bran extracts obtained from ethanol solvent (Et-SPT and Et-BB3-CMU) demonstrated greater suppression of the *SRD5A1-3* genes compared to extracts obtained from dichloromethane (Di-SPT and Di-BB3-CMU). Normally, tocopherols and *γ*-oryzanol, typically extracted with dichloromethane, are generally considered more effective inhibitors of *SRD5A* than polyphenols, which are predominantly extracted with ethanol [43]. This result could be explained by the concept of competitive binding to the 5 alpha reductase enzyme pockets. In fact, tocopherols and oryzanol have a strong affinity for SRD5A; the high concentrations of these compounds in the dichloromethane extracts may lead to competitive inhibition, where multiple molecules might have a binding competition to the enzyme [44]. The overabundance of tocopherols and *γ*-oryzanol in the dichloromethane extract might lead to a less effective inhibition because the binding sites on SRD5A become saturated or blocked by the less effective interactions. On the other hand, the polyphenols extracted with ethanol may act through different mechanisms or bind to different sites on the enzyme binding sites, resulting in more effective suppression of the *SRD5A1-3* genes [41,44,45]. Additionally, polyphenols have been shown to possess various bioactivities, including anti-inflammatory and antioxidant properties, which could contribute synergistically to their overall efficacy in gene suppression [14]. Further studies are required to explore the mechanisms of action and the potential synergistic effects between these compounds in inhibiting *SRD5A* and their implications for anti-hair loss.

The HFDPC cells are crucial for hair follicle development and the promotion of hair growth. As previously mentioned, the development of hair follicles needed the interaction between the fibroblast, mesenchymal, and epithelial cells, which receive signaling from HFDPC cells [46]. The Wnt/β-catenin (CTNNB1) signaling pathway is a key regulator in transitioning hair follicles from the telogen (resting) stage to the anagen (growth) stage. In this pathway, β-catenin plays a vital role in controlling cell metabolism and is crucial for maintaining hair follicles in the anagen stage. A decrease in β-catenin levels turns off the Wnt/β-catenin signaling pathway, leading to hair loss. Conversely, an increase in β-catenin levels activates this pathway, promoting HFDPC cell proliferation and migration, which supports hair growth [46]. The Sonic Hedgehog signaling pathway is a downstream process of the Wnt/β-catenin signaling pathway. This pathway plays a crucial role in signaling between mesenchymal and epithelial cells and significantly regulates their intracellular metabolism. Additionally, this pathway ultimately promotes hair follicle development, aids in repairing damage, and helps maintain the properties of hair follicle bulge stem cells [5]. In the Sonic Hedgehog signaling pathway, Sonic Hedgehog (SHH) ligands bind to the transmembrane receptor protein, or Patched (PTCH), which is a suppressor of the membrane protein smoothened (SMO). Afterward, SMO-free interacts with the Ellis van Creveld Syndrome (EVC) complex and moves to the primary cilia. After that, SMO induces glioma-associated oncogene (GLI) family transcription factors and protein kinase A (PKA) to form a macromolecular complex. Lastly, the GLI complex passes through the nucleus to initiate transcription of downstream target genes, leading to the transition (resting) stage to the anagen (growth) stage and inducing hair follicle development by stimulating the proliferation of HFDPC, mesenchymal, epithelial, and fibroblast cells in the anagen (growth) stage [5]. In addition, vascular endothelial growth factor is the essential mediator that controls blood vessel development and wound healing, leading to hair growth. Perifollicular vascularization is extensively active in the anagen (growth) stage and correlated with the stimulation of VEGF in follicular keratinocytes, resulting in the acceleration of hair growth. Moreover, the dimension of hair follicles and the diameter of the hair shaft were also increased by VEGF [47].

**Figure 4:**
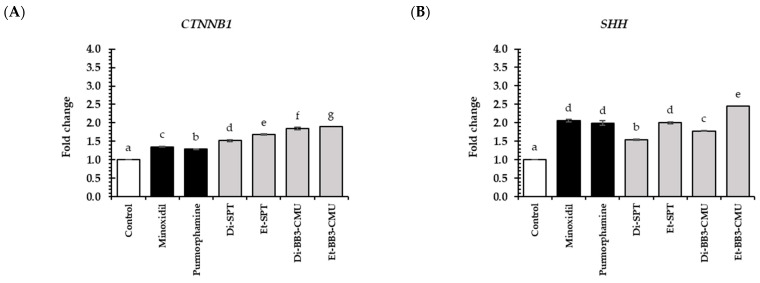

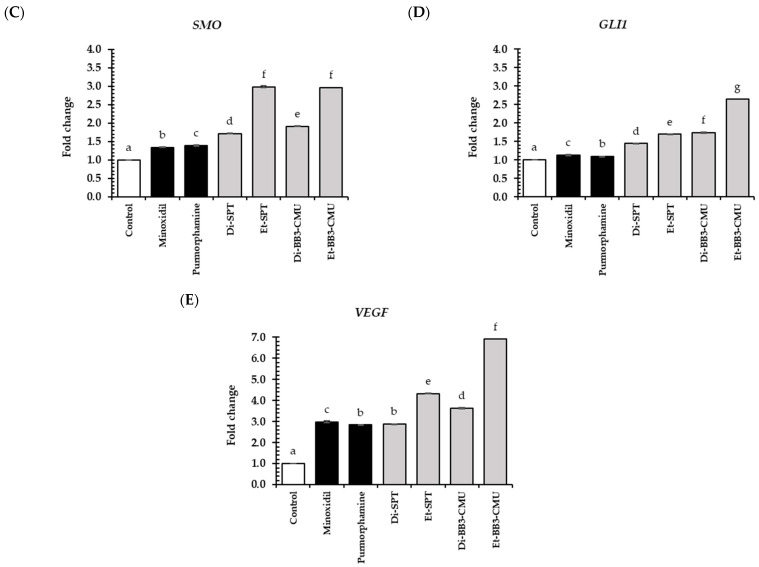
Effects of rice bran extracts and standard control (minoxidil and purmorphamine) (0.125 mg/mL) on the relative mRNA expression of genes associated with the Wnt/β-catenin signaling (**A**) *CTNNB1*; Sonic Hedgehog pathways (**B**) *SHH*; (**C**) *SMO*; (**D**); *GLI1*; and angiogenesis (**E**) *VEGF* in HFDPCs. Di-SPT: Sanpatong rice bran extracts using dichloromethane as the solvent; Et-SPT: Sanpatong rice bran extracts using ethanol as the solvent; Di-BB3-CMU: Buebang 3-CMU rice bran extracts using dichloromethane as the solvent; Et-BB3-CMU: Buebang 3-CMU rice bran extracts using ethanol as the solvent. Statistical analysis was performed using one-way ANOVA, followed by Tukey’s HSD test. Different letters (a–g) above the bars indicated significant differences (*p* < 0.05).

In the hair growth stimulation pathway, the Wnt/β-catenin signaling pathway (Figure 4A), the Sonic Hedgehog signaling pathway (Figure 4B–D), and the angiogenesis pathway (Figure 4E), all of the rice bran extract significantly expressed higher *CTNNB1*, *SHH*, *SMO*, *GLI1*, and *VEGF* expression compared to the untreated cells (control group) and standard controls (minoxidil and purmorphamine) (*p* < 0.05). For the Wnt/β-catenin pathway, the Et-BB3-CMU extract showed the highest fold change in *CTNNB1* expression of 1.90 ± 0.02, followed by the Di-BB3-CMU extract (1.85 ± 0.03) and Et-SPT extract (1.68 ± 0.02), respectively. For the Sonic Hedgehog pathway, the Et-BB3-CMU extract showed the highest fold change in all tested genes of *SHH* (2.44 ± 0.05), *SMO* (2.97 ± 0.01), and *GLI1* (2.65 ± 0.01), followed by Di-BB3-CMU and Et-SPT extract, respectively. Notably, Et-BB3-CMU extract exhibited the highest stimulation of *VEGF* expression (6.90 ± 0.01), followed by Et-SPT extract (4.33 ± 0.02) and Di-BB3-CMU (3.64 ± 0.02), respectively.

Remarkably, the ethanolic rice bran extracts (Et-SPT and Et-BB3-CMU) show the highest expression in all growth factor genes. This might be explained by the synergistic actions of other polyphenols in the extract. Previous studies have shown that polyphenols, such as caffeic acid and chlorogenic acid, may induce growth factor gene expression [7,48]. Additionally, our results showed that the ethanolic rice bran extracts outperformed the standard control, especially minoxidil, which is known to stimulate growth factor expression and its receptor in HFDPC cells [49]. Furthermore, the BB3-CMU extract showed the expression of the hair growth promotion gene and significantly higher activities than SPT extracts. This aligns with the significant differences observed in the bioactive compound levels, with BB3-CMU rice bran containing higher amounts of polyphenols, *γ*-oryzanol, and tocopherol than SPT rice bran (*p* > 0.05) [15].

## 3. Materials and Methods

### 3.1. Chemicals and Reagents

The Folin-Ciocalteu reagent, Triton X-100, aluminum chloride hexahydrate, and hydrogen peroxide were acquired from Merck (Darmstadt, Germany). The 2,2′-azino-bis (ethylbenzthiazoline-6-sulfonic acid) (ABTS), 2,2-diphenyl-1-picrylhydrazyl (DPPH), epigallocatechin gallate (EGCG), gallic acid, epigallocatechin gallate, 6-hydroxy-2,5,7,8-tetramethylchroman-2-carboxylic acid (Trolox), sulforhodamine B (SRB), diclofenac sodium, L-ascorbic acid, and dimethyl sulfoxide (DMSO) were obtained from Sigma Chemical (St. Louis, MO, USA). Thiobarbituric acid was purchased from VWR Chemicals (BDH Chem. Ltd., Poole, UK). Moreover, finasteride, dutasteride, and minoxidil were purchased from Wuhan W&Z Biotech (Wuhan, China). In the cell culture, the Follicle Dermal Papilla Cell Growth Medium Kits (catalog no. 2502) were obtained from Promo Cell GmbH (Heidelberg, Germany). The Roswell Park Memorial Institute 1640 Medium (RPMI-1640), Dulbecco’s Modified Eagle Medium (DMEM), Fetal bovine serum (FBS), antibiotic-antimycotic (100×), and penicillin-streptomycin solutions were purchased from Gibco Life Technologies (Thermo Fisher Scientific, Waltham, MA, USA). Griess reaction colorimetric kit was obtained from Invitrogen (Thermo Fisher Scientific, Inc., Eugene, OR, USA). All other chemicals used throughout the study were of analytical grade.

### 3.2. Plant Material and Crude Extracts

The Sanpatong (SPT) rice bran and Buebang 3 CMU rice bran (BB3-CMU) were obtained from Lanna Rice Research Center, Chiang Mai University, Chiang Mai, Thailand (18.758682743324353, 98.92977779492674), in March 2024. All rice bran were stored at the Pharmaceutical and Natural Products Research and Development Unit (PNPRDU), Faculty of Pharmacy, Chiang Mai University, with a voucher specimen number of PNPRDU65012 and PNPRDU65013, respectively. Then, both rice barn samples were dried at 60 °C for 48 h and subsequently ground through a 1 mm mesh. The subsequent step involved macerating 500 g of the sample powder in dichloromethane for 72 h for nonpolar extraction. The mixture was subsequently filtered through Watchman filter paper No. 1, and the extraction process was repeated twice. In order to perform polar extraction, the extracted material was macerated with 95% ethanol for 72 h and subsequently repeated twice. A combination of ethanolic compounds was filtered through Whatman No. 1 filter paper. The solvent was completely vaporized at a temperature of 40 °C using rotary evaporators. The crude extracts were stored at −20 °C until they were analyzed further.

### 3.3. Phytochemical Analysis and Antioxidant Activities

The total phenol and total flavonoid contents were determined in accordance with the methodology provided by Sangta et al. [50]. Folin-Ciocalteu reagent (60 μL) was added to the 30 μL samples, and 210 μL of 6.0% *w/v* saturated sodium bicarbonate was used. After that, the combined sample was maintained in darkness at room temperature for two hours. In consequence, the concentration of total phenol was determined by employing a UV-Vis spectrophotometer (Molecular Devices SpectraMax M3, Molecular Devices, CA, USA) at a wavelength of 725 nm. The total phenolic content was indicated as milligram gallic acid equivalents per gram of desiccated sample. Despite the fact that the total flavonoid content was high, the sample was mixed with 125 μL of distilled water and 7.5 μL of a 5.0% NaNO_2_ solution. The mixture was then left at room temperature for 5 min. Subsequently, 15 μL of a 10.0% AlCl_3_·6H_2_O solution was added, and the mixture was incubated for 6 min. After that, 50 μL of 1 M NaOH and 27.5 μL of distilled water were added. At a wavelength of 510 nm, the UV-Vis spectrophotometer was used to measure the absorbance of the test sample. The total flavonoid content was expressed in milligram catechin equivalents per gram of sample.

In this experiment, the measurement of antioxidants was conducted using three methods: DPPS, ABTS, and FRAP, as described by Wisetkomolmat et al. [15]. In DPPH 150 μL of freshly prepared DPPH solution (0.1 mmol/L) was combined with 50 μL of various doses of trolox (0.02–0.4 mg/mL) or extract solution. After 30 min of incubation at room temperature in the dark, absorbances at 515 nm were measured using a microplate reader (Molecular devices SpectraMax M3, USA). ABTS assay, ABTS was combined with 2.45 mmol/L potassium persulfate in a 2:1 ratio to produce a 7 mmol/L ABTS stock solution, which was subsequently stored in the dark for 16 h. The working solution was subsequently diluted with phosphate buffer (pH 7.4) to obtain an absorbance of 0.7 at 734 nm using a microplate reader. The results were reported in millimolar trolox equivalents per gram of extract. In term of FRAP, A working solution was created by combining acetate buffer (0.3 mol/L, pH 3.6), FeCl_3_ (20 mmol/L), and TPTZ solution (10 mmol/L in 0.04 mol/L HCl) in a 10:1:1 ratio. After adding 2.85 mL of the working solution to 150 μL of the diluted sample, absorbance was measured at 593 nm after 4 min. FRAP results were obtained as mmol/L ferric oxide equivalents per gram of sample by comparing absorbance variations in the test mixture to those caused by the Fe^2+^ concentration in the sample.

### 3.4. γ-Oryzanol and Tocopherol Analysis

The *γ*-oryzanol and tocopherol analyses were modified, followed by the Pestana-Bauer et al. [51] method. In order to assay *γ*-oryzanol, the samples were diluted in dichloromethane to a final concentration of 1 mg/mL and then filtered through a 0.45 μm syringe filter into a vial. The samples were determined using Shimadzu High-Performance Liquid Chromatography (HPLC) (Shimadzu, Kyoto, Japan) connected with Ultra C18 column (5 μm, 4.6 × 250 mm; Restek, Bellefonte, PA, USA) and a Shimadzu UV-Vis detector with a SPD-20A diode array detector. The methanol, acetonitrile, dichloromethane, and acetic acid in a ratio of 50:44:3:3 were used as mobile phase, with a flow rate of 1.4 mL/min. The uv detector was set to a wavelength of 330 nm.

Whereas the examination of tocopherol was carried out using Shimadzu HPLC apparatus, equipped with a fluorescence detector (RF-20A; Shimadzu Corporation, Kyoto, Japan), was used for the analysis. Reverse phase Ultra C18, 5 μm 250 × 4.6 mm column was used to separate tocopherols. Mixtures of acetonitrile, methenol, and isopropanol in the proportions of 50:40:10 (A) and 30:65:5 (B) constituted the mobile phase. The gradient program starts at 85% A for 15 min, then lowers to 10% A for 2 min. Subsequently, the percentage of A is increased to 50% for 5 min, and then to 85% for 3 min. At a discharge rate of 1 mL/min, the retention duration was 26 min. The fluorescence detector’s emission wavelength was 330 nm, whereas its excitation wavelength was 290 nm.

### 3.5. Polyphenol Profile Analysis

Extraction samples (10 mg) were diluted with 50% ethanol and filtered through a 0.45 µm nylon filter into a vial. The polyphenol profiles were analyzed in modified with Mighri et al. (2019) [52]. The assay was carried out using liquid chromatography (LC) (Agilent 1260 Infinity II series) and an electrospray ion (ESI) quadrupole mass spectrometer 6130 (Agilent Tech., Santa Clara, CA, USA). The Restek Ultra C18 column (250 × 4.6 mm, 5 μm, Restek, Bellefonte, PA, USA) was used for separation using reverse-phase column chromatography. The mobile phase contained A (0.2% acetic acid in 95% water and 5% methanol) and B (0.2% acetic acid in 50% water and 50% acetonitrile) with a linear gradient elution: 0–45 min, 10–20% B; 45–85 min, 20–55% B; 85–97 min, 55–100% B; 97–110 min, 100% B; the initial conditions were held for 10 min as a re-equilibration step. The mobile phase had a flow rate of 0.5 mL per minute. The injection volume was 20 μL, and the column temperature was kept at 40 °C. Full scan spectra from 100 *m/z* with 250 ms/spectrum were operated in the negative selected ion monitoring (SIM) as follows: a capillary voltage of −3.5 V, a flow rate of 1.5 L/min of nebulizing gas, a flow rate of 12 L/min of dry gas (N2), a temperature of 250 °C for the DL (dissolving line), a temperature of 400 °C for the block source, a fragmentor voltage of 70 V, and a capillary voltage of −3.5 V. Software from Agilent Technologies, Santa Clara, CA, USA, called OpenLab version A.01.10.128 was used to process the spectra.

### 3.6. In Vitro Cell Viability and Proliferation Assay

The human prostate cancer (DU-145) and macrophage (RAW 264.7) cells were obtained from the American Type Culture Collection (Rockville, MD, USA). DU-145 cells were cultured in RPMI-1640 culture medium supplemented with 10% FBS and a 1% penicillin/streptomycin (100×) solution. While RAW 264.7 cells were grown in DMEM culture medium supplemented with 10% FBS and a 1% penicillin/streptomycin (100×) solution. Additionally, the human hair follicle dermal papilla cells (HFDPCs) were purchased from Promo Cell GmbH, Heidelberg, Germany, and grown in Follicle Dermal Papilla Cell Growth Medium Kits, supplemented with a 1% antibiotic-antimycotic (100×) solution.

The sulphorhodamine B (SRB) assay was utilized to determine the cell viability and proliferation of rice bran extract (Di-SPT, Et-SPT, Di-BB3-CMU, and Et-BB3-CMU) and standard controls (diclofenac sodium, dutasteride, finasteride, and minoxidil) in a concentration ranging from 0.0625 to 2 mg/mL on DU-145, RAW 264.7, and HFDPC cells [41]. In brief, the cells at the concentration of 1 × 10^5^ cells/mL were seeded to a 96-well plate and cultured for 24 h at 37 °C with 5% CO_2_. After that, the cells were exposed to the rice bran extracts and the standard control for 24 h. Then, the cells were fixed with 50% (*w*/*v*) trichloroacetic acid (TCA) at 4 °C for 1 h and stained with 0.04% (*w*/*v*) SRB solution for 30 min. Finally, the bound dye was removed by 10 mM Tris base, and the absorbance was read at 515 nm. The concentrations above 80% cell survival were selected for further experiments. The percentage of cell survival was calculated using the following Equation (1):

(1) $$Cell\;viability\;(\%)=\frac{\left({Absorbance}_{\;sample}-{Absorbance\;}_{blank}\right)}{\left({Absorbance}_{\;control}-{Absorbance\;}_{blank}\right)}\times 100$$

### 3.7. Anti-Inflammatory Activity Assay

The level of nitrite production that accumulated in the cell culture supernatants was determined by the Griess reaction colorimetric assay kit. Briefly, the RAW 264.7 and HFDPC cells were added (1 × 10^5^ cells/mL) and cultured for 24 h at 37 °C with 5% CO_2_. After that, the cells were pre-exposed with 0.125 mg/mL of rice bran extract (Di-SPT, Et-SPT, Di-BB3-CMU, and Et-BB3-CMU), standard control (diclofenac sodium), and incomplete medium (blank) for 2 h. Then, the cells were exposed to 0.001 mg/mL of LPS for 24 h. After incubation, the cell culture supernatants were collected to react with the Griess reagent solution according to the manufacturer’s recommendations. The calibration standard was plotted from the absorbance of standard nitrite at a concentration of 0.01 to 100 µM [53].

### 3.8. Thiobarbituric Acid-Reactive Substances (TBARS) Assay

The antioxidant activity of rice bran extract (Di-SPT, Et-SPT, Di-BB3-CMU, and Et-BB3-CMU), standard control (L-ascorbic acid), and the control group (incomplete medium) were evaluated using the TBARS assay. In short, HFDPC cells were seeded to 6-well plates (1 × 10^5^ cells/mL) and cultured for 24 h at 37 °C with 5% CO_2_. Afterwards, the cells were pre-exposed with 0.125 mg/mL of sample for 24 h, followed by post-treatment with H_2_O_2_ for 2 h. Then, the cells were collected and reacted with a mixture solution (1% Triton X-100, 0.6% thiobarbituric acid, and 15% trichloroacetic acid) at 100 °C for 10 min. Finally, the cells were cooled down in the freezer (−80 °C) for 10 min. The final product of lipid peroxidation was measured at 532 nm. The level of MDA in HFDPC cells was evaluated in comparison to the control group [53].

### 3.9. Semi-Quantitative Reverse Transcription and Polymerase Chain Reaction Analysis

Gene expression level in the androgen pathway (*SRD5A1-3*) and the genes associated with hair growth promotion, such as Wnt/β-catenin (*CTNNB1*), Sonic Hedgehog (*SHH*, *SMO*, and *GLI1*), and angiogenesis pathways (*VEGF*), was determined in DU-145 and HFDPC cells as previously described [42]. The rice bran extracts (Di-SPT, Et-SPT, Di-BB3-CMU, and Et-BB3-CMU) were compared to the standard controls (dutasteride, finasteride, purmorphamine, and minoxidil) at the same concentration of 0.125 mg/mL. The RNA extraction was determined using the E.Z.N.A.^®^ Total RNA Kit I (Omega BioTek, Norcross, GA, USA). The concentration of total RNA was determined using the Qubit^TM^ RNA HS Assay Kit (Invitrogen, Carlsbad, CA, USA) and the NanoDrop Spectrophotometers (Thermo Fisher Scientific, Waltham, MA, USA). The expression of the gene was evaluated using the MyTaq^TM^ One-Step RT-PCR Kit (Bioline, Memphis, TN, USA). The primer sequences are indicated in Table 5. Glyceraldehyde 3-phosphate dehydrogenase, or *GAPDH*, was utilized to normalize the expression of the target genes. The results were demonstrated as fold changes in gene expression. Finally, agarose gel electrophoresis was used to detect PCR products. The gel images and band intensity were determined by the Gel Doc™ EZ System (Version 3.0; Bio-Rad Laboratories, Hercules, CA, USA) and Image Lab™ software (Version 5.1).

### 3.10. Statistical Analysis

The result was indicated as the mean ± standard deviation (SD). Statistical analysis was determined using SPSS 23.0 Software (SPSS Inc., Chicago, IL, USA) with a one-way ANOVA followed by Tukey’s test. Statistical significance was evaluated as a *p*-value below 0.05.

## 4. Conclusions

Our research demonstrated that the by-product derived from the bran of Buebang 3 CMU extracts contained significantly higher levels of bioactive compounds, including *γ*-oryzanol, tocopherols, and various polyphenols such as phytic acid, ferulic acid, and chlorogenic acid, compared to Sanpatong extracts. Additionally, the ethanolic extracts exhibited greater levels of bioactive compounds and antioxidant activities than those extracted with dichloromethane. Furthermore, the ethanolic Buebang 3 CMU extract (Et-BB3-CMU) promoted the proliferation of human hair HFDPCs, supporting hair growth during the anagen phase of the hair cycle. Et-BB3-CMU also demonstrated antioxidant and anti-inflammatory activities by reducing TBARS levels and nitrite production in HFDPCs, which contributes to decreased inflammation and oxidative damage in hair follicles. Furthermore, it exhibited significant in vitro anti-hair loss properties by inhibiting gene expression levels in the androgen pathway (*SRD5A1-3*) in both DU-145 and HFDPC cells, while upregulating hair growth-promoting genes such as Wnt/β-catenin (*CTNNB1*), Sonic Hedgehog (*SHH*, *SMO*, *GLI1*), and angiogenesis-related factors (*VEGF*) specifically in HFDPC cells. Both varieties of rice bran and the extraction solvent significantly influence the bioactive activities. Understanding these interactions is essential for refining the extraction process to enhance the therapeutic potential of the bioactive compounds in rice bran for hair growth promotion and anti-hair loss treatment. Therefore, further study is required to clarify the molecular mechanisms of each biological compound in rice bran to better understand the application of Et-BB3-CMU as a promising active ingredient for hair loss prevention and growth promotion.

## Figures and Tables

**Table 5:** Specific primer sequences used for semi-quantitative RT-PCR.

Target Pathway	Primer Name	Gene Bank No.	Type of Sequence	Primer Sequence (5′-3′)	Annealing Temperature (°C)
Internal control	*GAPDH*	NM_001289745.3	Forward	GGAAGGTGAAGGTCGGAGTC	55
			Reverse	CTCAGCCTTGACGGTGCCATG	
5α-reductase	*SRD5A1*	NM_001047.4	Forward	AGCCATTGTGCAGTGTATGC	52
			Reverse	AGCCTCCCCTTGGTATTTTG	
	*SRD5A2*	NM_000348.4	Forward	TGAATACCCTGATGGGTGG	52
			Reverse	CAAGCCACCTTGTGGAATC	
	*SRD5A3*	NM_024592.5	Forward	TCCTTCTTTGCCCAAACATC	50
			Reverse	TCCTTCTTTGCCCAAACATC	
Wnt/β-catenin	*CTNNB1*	NM_001330729.2	Forward	CCCACTAATGTCCAGCGTTT	55
			Reverse	AACCAAGCATTTTCACCAGG	
Sonic Hedgehog	*SHH*	NM_000193.4	Forward	AAAAGCTGACCCCTTTAGCC	51
			Reverse	GCTCCGGTGTTTTCTTCATC	
	*SMO*	NM_005631.5	Forward	GAAGTGCCCTTGGTTCGGACA	57
			Reverse	CCGCCAGTCAGCCACGAAT	
	*GLI1*	NM_005269.3	Forward	GCAGGGAGTCAGCCAATACAG	56
			Reverse	GAGCGGCGGCTGACAGTATA	
Angiogenesis	*VEGF*	NM_001025366.3	Forward	CTACCTCCACCATGCCAAGT	55
			Reverse	GCGAGTCTGTGTTTTTGCAG	

## Data Availability

The original contributions presented in the study are included in the article, further inquiries can be directed to the corresponding authors.
